# Pleomorphic Adenoma of the Parotid Gland and the Parapharyngeal Space: Two Diametrically Opposing Surgical Philosophies for the Same Histopathologic Entity? †

**DOI:** 10.3390/jcm11010142

**Published:** 2021-12-28

**Authors:** Benita Hornung, Jannis Constantinidis, Vivian Thimsen, Abbas Agaimy, Michael Koch, Antoniu-Oreste Gostian, Matti Sievert, Sarina Katrin Müller, Heinrich Iro, Konstantinos Mantsopoulos

**Affiliations:** 1 Department of Otorhinolaryngology, Head and Neck Surgery, Friedrich-Alexander-Universität Erlangen-Nürnberg (FAU), 91054 Erlangen, Germany; benita.hornung@gmx.de (B.H.); vivian.thimsen@uk-erlangen.de (V.T.); michael.koch@uk-erlangen.de (M.K.); antoniu-oreste.gostian@uk-erlangen.de (A.-O.G.); matti.sievert@uk-erlangen.de (M.S.); Sarina.mueller@uk-erlangen.de (S.K.M.); heinrich.Iro@uk-erlangen.de (H.I.); 2 1st Deparment of ORL, Head & Neck Surgery, Aristotle University of Thessaloniki, 54636 Thessaloniki, Greece; janconst@otenet.gr; 3 Institute of Pathology, Friedrich-Alexander-Universität Erlangen-Nürnberg (FAU), 91054 Erlangen, Germany; abbas.agaimy@uk-erlangen.de

**Keywords:** pleomorphic adenoma, parotid gland, parapharyngeal space, capsule, satellite, pseudopodium

## Abstract

Background: The aim of this study was to investigate the histopathologic findings in parotid and parapharyngeal pleomorphic adenomas and draw conclusions concerning the surgical strategy. Methods: Retrospective study of medical charts of patients with resected pleomorphic adenomas (PA) between 2005 and 2020 at two tertiary medical referral centers. Histologic specimens were reexamined by an experienced head and neck pathologist. Patients with insufficient/incomplete data were excluded from our study sample. Results: A total of 844 patients formed our study sample (291 men, 553 women, average age 48.9 years); 786 cases had a PA in the parotid gland (PG) (93.1%), and the remaining 58 cases had a PA in the parapharyngeal space (PS) (6.9%). Recurrences were detected in 8/844 cases (7/786 in the PG, 1/58 in the PS, 0.94% in total) with a mean follow-up time of 86.7 months (10–189 months) with no statistically significant differences between the study groups (*p* = 0.527). Our analysis showed that parapharyngeal pleomorphic adenomas are characterized by a lower incidence of an intact anatomical capsule (71.4% vs. 82.6%, *p* = 0.035) and a remarkably more frequent occurrence of satellite nodules (20.7% vs. 7.5%, *p* < 0.001). Conclusions: The more challenging histopathologic profile of parapharyngeal pleomorphic adenomas points towards the fact that parapharyngeal surgery should remain in the hands of experienced surgeons at high-volume centers.

## 1. Introduction

Understanding the histologic characteristics of the tumor capsule is fundamental in the management of PA [1]. Various histologic subtypes [2], several degrees of capsular thickness [3] and intactness [4], pseudopodia, and satellite nodules at a variable distance from the main lesion [5] are factors that constitute a challenging surgical profile of this lesion. Completeness of resection presupposes consideration of all the aforementioned characteristics of the capsule, so that the principle “do not see the capsule wherever possible” seems to summarize the mainstay of surgical treatment [6]. Even though the association of the surgical method and the mechanism of recurrence in pleomorphic adenomas (PA) of the parotid gland (PG) is doubted in the relevant literature, a certain degree of interdependence between the two factors can hardly be dismissed [7,8,9]. Abandoning the two edges of surgery (enucleation and complete parotidectomy in every case) led to a completely acceptable rate of postoperative complications and a drastic decrease in the long-term (e.g., 20 years’ follow-up) recurrence rate in 5–7% [10].

Currently, there is much controversy in the relevant literature regarding the ideal surgical management of pleomorphic adenomas [11]. Despite the fact that numerous literature reports propose strict adherence to the surgical rule “avoid seeing the tumor capsule wherever possible” in PAs of the PG^6^, the most common approach in the management of PAs in the parapharyngeal space traditionally consists in a transcervical rather blunt dissection along the capsule of the lesion, which is equal to the so-called enucleation in the PG. Remarkably, two diametrically opposing surgical philosophies for the same histopathologic entity seem to share comparable oncologic outcomes [12,13,14,15,16]. This realization gave rise to several questions that are hidden behind the somewhat provocative title of this article: Do PAs of the PG and PS really belong to the same histologic entity? If so, why do different approaches lead to a similar outcome? Is the role of the capsule in the management of PAs perhaps somewhat overestimated? Are we perhaps still far from understanding the actual mechanism of pleomorphic adenoma recurrence?

In trying to find an answer to the aforementioned discrepancy (absence of effect of a completely different surgical philosophy in the management of the same histologic entity in different locations of the same organ) and our observations, we examined both the clinical and the histopathologic records of patients with PAs comparatively at two academic centers. Evidence of potential histopathologic differences in these tumors could thus have significant implications for the surgical method.

## 2. Materials and Methods

This study was performed at two academic tertiary referral centers specializing in salivary gland diseases (Department of Otorhinolaryngology, Head and Neck Surgery, University of Erlangen–Nuremberg, Erlangen, Germany and Department of Otorhinolaryngology, Head and Neck Surgery, University of Thessaloniki, Thessaloniki, Greece). For this study, an experienced head and neck pathologist (A.A.) critically reevaluated the histologic slides of the pathologic specimens of all patients who were treated for PAs of the PG and the PS between 2005 and 2020 (inclusion criterion). The exclusion criteria were as follows: patients with insufficient clinical data as well as all cases with PAs, in whom the whole periphery of the tumor was not histologically ascertainable on at least two slides based on the tumor size.

For the purposes of the histopathologic analysis, PAs were divided into 3 histologic subtypes on the basis of the stroma-cell proportion, according to the classification of Seifert et al. [2]: the classic subtype (stroma content of 30% to 50%), the stroma-rich (myxoid) subtype (stroma content of more than 50%), and the cellular subtype (stroma content of 30% or less). Furthermore, we paid special attention to the specific characteristics of the capsule likely to be associated with the potential of recurrence, such as the presence of a complete and intact tumor capsule, surgically induced capsular defects, pseudopodia, and satellite nodules. For the purpose of comparison with the relevant literature, we used the nomenclature proposed by Zbaeren et al. [4]: a complete capsule assumed complete encapsulation of the tumor tissue in an anatomically intact capsule. A pseudopodium assumes a protrusion of the main tumor localized within the main tumor capsule. Finally, satellite nodules are distinct tumor nodules near the main tumor but outside the main tumor capsule, separated from it by salivary or fat tissue without any connection to the main tumor.

The aim of our study was to compare the cases with PAs of the PG and PS in terms of epidemiologic factors (age, gender) as well as the incidence of the aforementioned histopathologic parameters. Moreover, our study cases were compared with regard to several satellite nodule-specific characteristics (number of satellite nodules, distance of satellite nodule from main lesion). Statistical analysis was performed using the t-test for the epidemiologic analysis, the x^2^ test, as well as the t-test for the comparison of histologic parameters with 95% confidence intervals (CIs). The software SPSS version 21 for Windows (SPSS, Inc., Chicago, IL, USA) was used for the analysis. A *p*-value of <0.05 was considered statistically significant. The Institutional Review Board (IRB) of the University Hospital of Erlangen approved this study.

## 3. Results

A total of 844 patients formed our study sample (291 men, 553 women, male to female ratio: 0.53:1). Of these, 786 cases had a PA in the PG (93.1%), and the remaining 58 cases had a PA in the PS (6.9%). Mean age was 48.9 years (±15.4, range: 12–87 years). The group of PAs in the PG consisted of 273 men and 513 women with a mean age of 48.7 years (±15.4, range: 12–87 years). The group of PAs in the PG consisted of 18 men and 40 women with a mean age of 50.2 years (±14.0, range: 25–84 years). No statistically significant differences were detected between the study groups regarding gender (*p* = 0.567) or age (*p* = 0.481). Recurrences were detected in 8/844 cases (7/786 in the PA, 1/58 in the PS, 0.94% in total) with a mean follow-up time of 86.7 months (10–189 months), with no statistically significant differences between our study groups (*p* = 0.527, Table 1). Interestingly, the mean surgical experience of the surgeons who managed the PAs of the PS was 17.1 years (4–32 years). The mean surgical experience of the surgeons who operated on the PG group was 11.2 years (2–32 years). The PAs of the PS were significantly larger (40.5 ± 13.8 mm) in comparison to their parotideal counterparts (23.7 ± 10.6 mm, *p* < 0.001). Two cases had a history of radiation exposure in childhood. In three cases of PA in the PG, an additional synchronous (two cases with cystadenolymphoma and in one case basal cell adenoma) was present and in one case of the same group a metachronous tumor (cystadenolymphoma) appeared.

**Figure 1:**
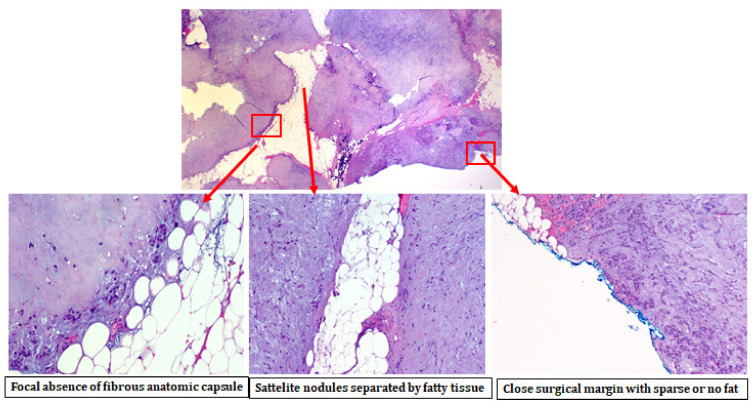
Pleomorphic adenoma of the parapharyngeal space with focal absence of the fibrous capsule, presence of satellite nodules, and a close surgical margin with sparse or no presence of fatty tissue.

Our comparative analysis showed that parapharyngeal pleomorphic adenomas are characterized by a lower presence of an intact anatomical capsule and a remarkably more frequent occurrence of satellite nodules (Figure 1, Table 2). Table 3 shows the comparative analysis of several satellite nodule-specific characteristics (number, distance from main lesion) between the two study groups.

## 4. Discussion

**Figure 2:**
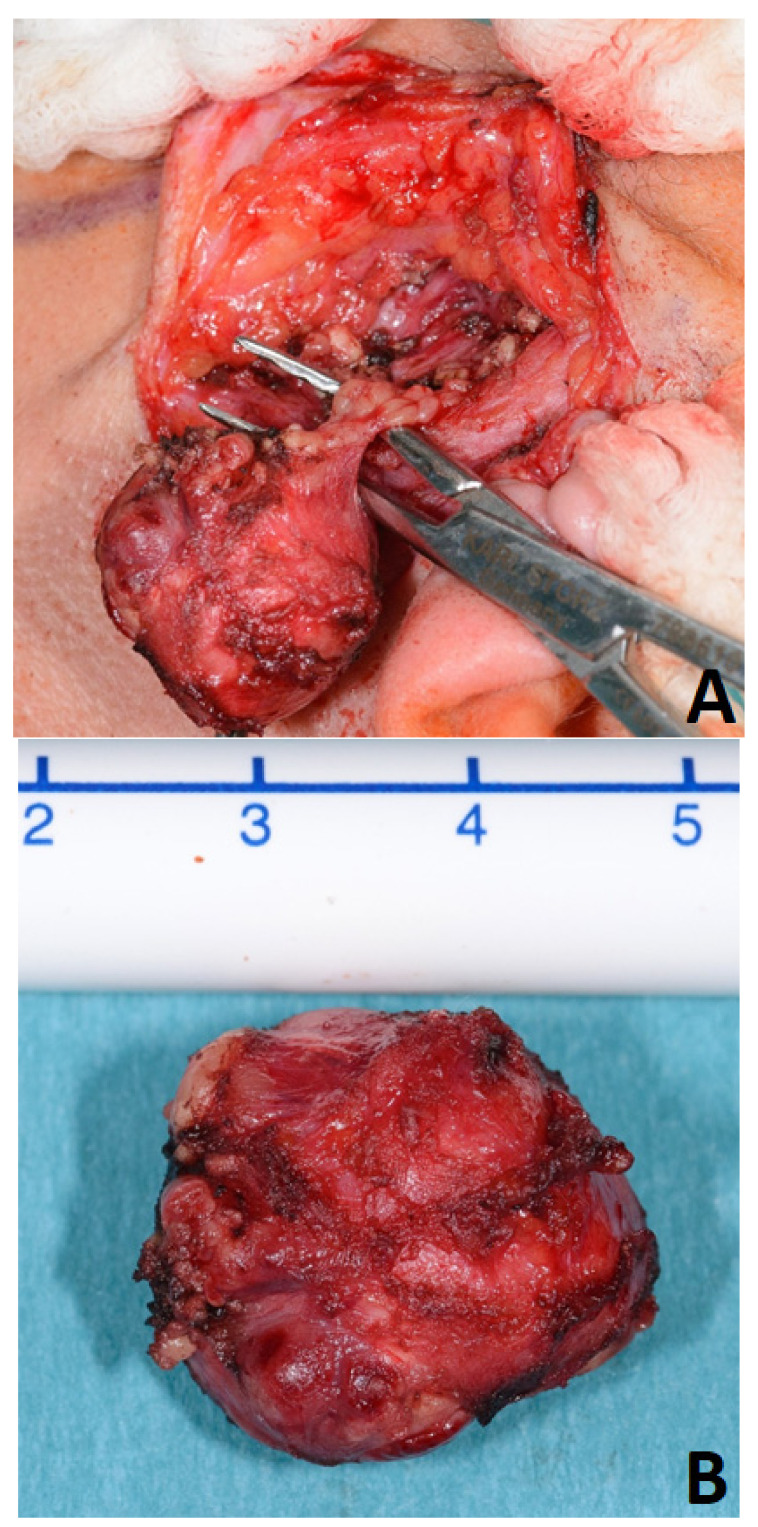
Extracapsular dissection (**A**) of a pleomorphic adenoma of the parotid gland (**B**).

**Figure 3:**
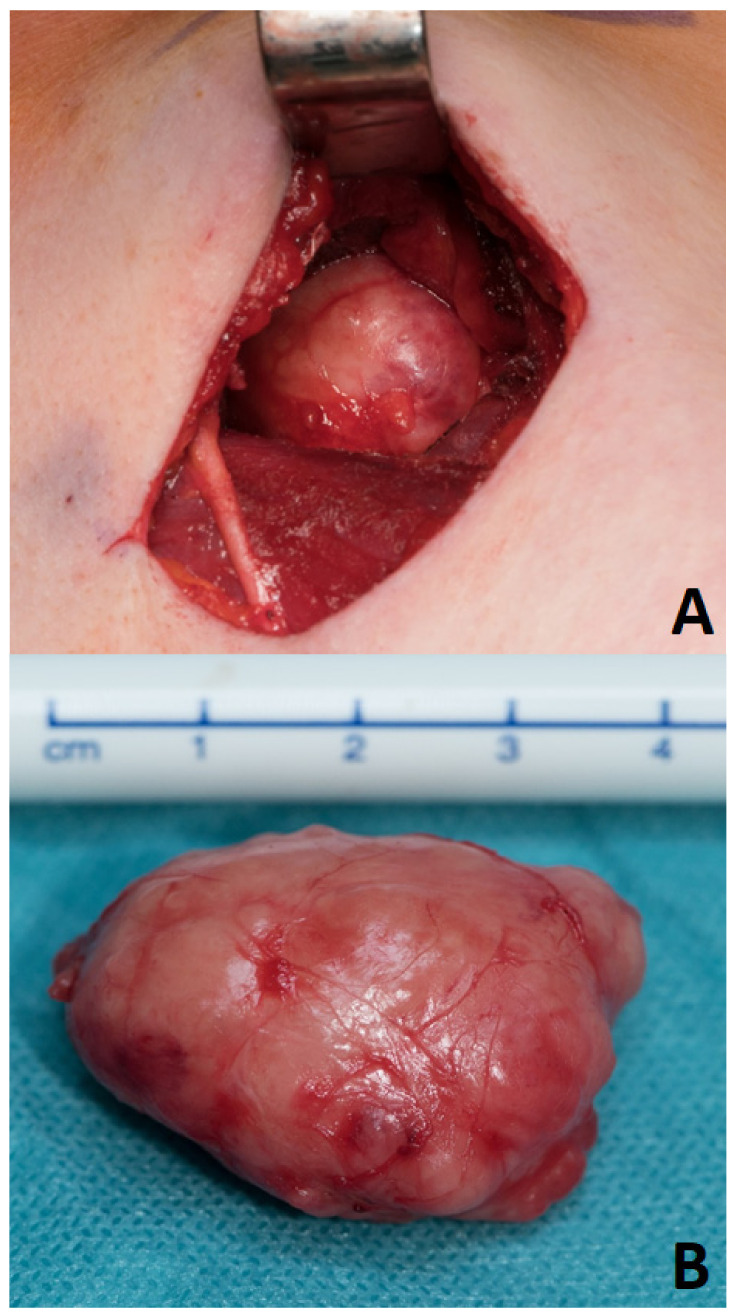
Parapharyngeal space: Transcervical capsular resection (**A**) of a pleomorphic adenoma (**B**).

For many decades, parotid surgery was dominated by the dogma that only a dissection of the facial nerve in every case without exception could warrant completeness of resection of the PA and sufficient control over the functional integrity of the facial nerve [17]. Every effort towards reducing surgical invasiveness, e.g., avoiding nerve exposure and surgical dissection around the tumor with preservation of a cuff of healthy tissue around it (“extracapsular dissection”, Figure 2), was not received with open arms and encountered skepticism among several working groups [1,17,18]. Remarkably, this otherwise highly controversial surgical modality is accepted as one of the most common and undoubtedly least invasive ways of managing the same lesions in the parapharyngeal space [15,19,20,21,22]. In fact, extracapsular dissection in the parapharyngeal space tends to take the form of capsular dissection (or extracapsular enucleation [23]) around a large amount of surface of the PA. The limited accessibility of a tumor hidden behind the mandible at a small distance from the skull base, the adhesion to the pterygoid muscles, and the mandible entail a blunt dissection around (the most part of) the lesion. In this hardly accessible space, transcervical resection can mostly be performed only by means of the surgeon’s finger, a peanut swab holding forceps, or a periosteal elevator, without any possibility of avoiding a broad exposure of the capsule (Figure 3) [15].

According to the current state of anatomic knowledge, there is no anatomic landmark that formally separates the PG into two lobes, as there is no soft tissue dissection plane between them. For surgical reasons, the sagittal plane of the facial nerve serves as a fictive border between a superficial and a deep lobe of the PG. The specific topographic anatomy of the deep lobe is responsible for several tumor pathway patterns [16,24]: a PA can grow either in the lateral direction, pushing the facial nerve outwards, or through the “stylomandibular tunnel” (demarcated through the skull base cranially, the stylomandibular ligament medially and the ascending ramus of the mandible laterally, as described by Patey and Thackray [25]) into the parapharyngeal space, in this case taking an “hourglass” or dumbbell-shaped tumor form. Medial to the mandible, the PA seems to follow the path of least resistance and can easily occupy a large portion of the PS.

In trying to explain the significantly higher incidence of incomplete capsule and satellite nodules in parapharyngeal PAs, we should consider the two dominant theories concerning formation of the PA capsule. According to the first, the capsule already exists at the onset of the development of the PA. A more aggressive behavior of the tumor parenchyma against the capsule, with a varying “subtype-dependent” ingrowth of tumor material in the capsule, leads to a thinning of the capsule with the creation of defects as the tumor grows in size [26]. The lack of difference in the distribution of the histologic subtypes between our study groups (*p* = 0.991) weakens the argumentation in favor of this concept. The second theory, presented by Cotran et al. [27], Evans [28], and Eneroth [29], intends the capsule rather to be a product of the host tissue and describes the formation of a thin pseudocapsule (and later on a thicker one) as “reactive” connective tissue around the expanding PA. If we adopt this theory, we have to investigate the properties of the host tissue in the PG and the PS. The PAs of the PG develop in a firm, hardly dilatable space between rigid structures such as the skin, firm parotid fascia, and the ramus of the mandible inside a non-stretchable parenchyma with multiple fibrous septa. The high local tissue pressure in the glandular parenchyma may lead to the formation of a stronger capsule and generally impede the development of satellite tumors. In contrast to this, the PS contains muscle fibers, lots of loose areolar tissue, as well as fatty tissue and only sparse glandular parenchymal. Medially, the pharyngeal wall does not pose any resistance to further growth and is easily displaced medially, as shown by voluminous lesions with displacement of the lateral oropharyngeal wall. Similarly, the pterygoid muscles are compressible and smooth and allow further growth of these lesions without posing any significant resistance. Acceptance of this potential interaction between the local environment and the tumor could offer sufficient explanation for the more well-defined and stable capsule of PAs in the PG and a less well-defined, rather ill-margined capsular profile in their parapharyngeal counterparts, as shown in our analysis.

A second point of significant difference between our study groups lay in the incidence of satellite nodules. This characteristic presented far more frequently in parapharyngeal lesions and points to an already described potential association between an incomplete capsule and satellite nodules [30]. In contrast, no differences could be detected between our study groups in the incidence of pseudopodia or specific characteristics of the satellite nodules. In a previous literature report of the same working group, it was postulated that, over the course of time, tumor material builds a projection still inside the capsule (onset of pseudopodium), separates itself from the parenchyma with fibrous tissue still remaining enclosed within the capsule (“mature” pseudopodium), slowly penetrates the capsule of the PA, and leaves the tumor taking a part of the capsule with it (satellite nodule), leaving a capsular defect behind in the main lesion [30]. If we accept the pseudopodium as a precursor of a satellite nodule, our findings lead to the conclusion that the majority of PAs of the PS could have apparently reached a “mature” situation by occupying a huge part of the parapharyngeal space at initial presentation without an ongoing formation of satellite nodules.

A thorough examination of the relevant literature revealed only one article dealing with an association between localization within the PG and capsular characteristics: Eighteen years ago, Harney et al. examined 31 pleomorphic adenomas and found a thicker capsule with fewer defects in deep lobe compared to superficial lobe lesions [3]. In this article, the word “parapharyngeal” does not appear at all, so that possibly only PAs from the portion of the deep lobe lateral to the mandible were examined. Accordingly, the rigid, hardly expandable space between the mandible and the hardly stretchable facial nerve could explain the better-developed capsules in the “deep lobe”. Based on this, Harney suggested that deep lobe tumors (as defined in this article) could be removed by means of enucleation, without the risk of opening of the tumor and spillage of material in the surgical wound [3]. Under the aforementioned precondition, this report supports our explanation for the different capsular profiles of PA in the PG and the PS.

A further part of our analysis showed that the mean size of PAs of the PS was larger by 41.2% than that of their parotid counterparts. This finding could be easily explained by the common experience that parapharyngeal lesions are characterized by an asymptomatic growth and are mostly diagnosed in an imaging examination for other reasons [21,22]. In another literature report of the same working group, a positive correlation between PA size and the incidence of satellite nodules could be detected [5]. The question arising from the combination of these findings would be to what extent the higher incidence of satellite nodules in the parapharyngeal PS could be attributed to their larger size itself and not to the specific tissue conditions of each anatomic space. In trying to investigate this issue, we compared the subgroups of PAs with a size > 25 mm (mean size of all study cases, see Table 1) for incidence of incomplete capsule and satellite nodules in both localizations. In the subgroup of these larger lesions, parapharyngeal PAs preserved their superiority regarding incidence for both examined histologic parameters (*p* < 0.001 and *p* = 0.018 respectively). This finding strengthens the role of the tissue environment specific conditions over the effect of lesion size on the histologic profile of the PAs. Considering further potential explanations for our findings, we reviewed the relevant literature for information on the molecular characteristics and microenvironment of PA. The elucidation of the molecular landscape of PAs has been rather inadequate up to now. Fusion genes, such as PLAG1 and HMGA2, are supposed to be involved in the pathogenesis of this lesion [31,32]. Regarding a potential effect of the PA microenvironment on the biologic behavior of the lesion, a series of reports deal with the remodeling of the cytoskeleton of the tumor leading to complex patterns of microscopic intralesional differentiation (cellular adaptation) [33] without any phenotypic changes in the periphery of the lesion. Concerning immune microenvironment, this is rather relevant to the malignant transformation of this entity and does not seem to affect the biologic behavior of the PA [34].

For the sake of correctness, the retrospective nature of the study, the variable number of histological slides per case (based on tumor size) as well as the moderate duration of follow-up (particularly for pleomorphic adenomas) should be regarded as potential limitations of the present study. Additionally, the limited follow-up of the study (86.7 months) could affect the number of recurrences in the study cohort. Moreover, the difference in the number of patients diagnosed with PA of the PG and PS, although it reflects the clinical reality, should be characterized as a further drawback of this study

## 5. Conclusions

The aim of our study was to examine the behavior of parotideal pleomorphic adenoma lesions in two completely different environments (in terms of rigidity of the surrounding tissue elements) and to draw conclusions concerning the implications of surgical treatment. In contrast to the general convincement that a thicker and more stable capsule of the parapharyngeal lesions makes spillage significantly less likely to occur and allows pronounced dissecting manipulations around the tumor^3^, our study could not offer any data to support this. Our findings could be explained by the lower rigidity and higher grade of freedom for expansion in the PS and point towards the fact that parapharyngeal surgery should be performed only by experienced, high-volume surgeons with awareness of the demanding histologic profile of PAs in this localization. It seems that PAs of the PG and PS are two different phenotypes of the same histologic entity. Capsular dissection in the parapharyngeal space seems to be somewhat facilitated through the limited number of satellite lesions and their limited distance to the main lesion. A thorough review of the relevant literature concerning a potential effect of the molecular landscape and tumor microenvironment could not reveal any association between these parameters and the capsular phenotype as well as the biologic behavior of the PA in different anatomic sites. The lack of such information on the clinical relevance of these factors at present and necessitates further experimental studies for elucidation of potential molecular pathogenetic pathways.

## Figures and Tables

**Table 1:** Comparative analysis of the demographic and clinical parameters in pleomorphic adenomas of the parotid gland and parapharyngeal space in our study (*p* values in bold: statistically significant).

	Parotid Gland	Parapharyngeal Space	Total	*p* Value
Number of cases	786 (93.1)	58 (6.9)	844 (100)	-
Age (years)	48.7 (±15.4, range: 12–87)	50.2 (±14.0, range: 25–84)	48.9 (±15.4, range: 12–87)	0.481
Gender (male/female)	273/513	18/40	291/553	0.567
Size (mm)	23.7 (± 10.6, range: 4–105)	40.5 (± 13.8, range: 18–75)	24.9 (±11.6, range: 4–105)	**<0.001**
Recurrences (%)	7/786 (0.8)	1/58 (1.7)	8/844 (0.9)	0.527
Average time from diagnosis to recurrence (months)	72.8 (47–97)	138	82.1 (47–138)	-

**Table 2:** Comparative analysis of the histopathologic parameters in pleomorphic adenomas of the parotid gland and parapharyngeal space in our study (*p* values in bold: statistically significant).

Histopathologic Features		Parotid Gland (%)	Parapharyngeal Space (%)	Total (%)	*p*
Histologic subtype [2]	Hypercellular subtype (stroma content < 30%)	189 (24)	14 (24.1)	203	0.972
	Mixed subtype (stroma content 30–50%)	220 (28)	17 (29.3)	237	
	Stroma-rich (myxoid) subtype (stroma content > 50%)	377 (48)	27 (46.6)	404	
	Total	786 (100)	58 (100)		
Integrity of capsule	No capsule at all	32 (4.1)	4 (6.9)	36	**0.035**
	Partial encapsulation	105 (13.3)	14 (24.1)	119	
	Intact capsule	649 (8.3)	40 (69)	689	
	Total	786 (100)	58 (100)		
Pseudopodia	Presence of pseudopodia	343 (43.6)	22 (37.9)	365	0.397
	No pseudopodia	443 (56.4)	36 (62.1)	479	
	Total	786 (100)	58 (100)		
Satellite nodules	Presence of satellite nodules	59 (8.1)	12 (20.7)	71	<0.001
	Absence of satellite nodules	727 (92.5)	46 (79.3)	773	
		786 (100)	58 (100)		
Surgical invasion of the capsule	Surgical invasion of the capsule	33 (4.2)	5 (8.6)	38	0.117
	No surgical invasion of capsule	753 (95.8)	53 (91.4)	806	
	Total	786 (100)	58 (100)		

**Table 3:** Comparative analysis of the satellite nodule-specific histopathologic parameters in pleomorphic adenomas of the parotid gland and the parapharyngeal space in our study.

	Parotid Gland	Parapharyngeal Space	*p*
Number of satellite nodules/lesion (±SD)	1.32 (±0.6)	1 (±0.0)	0.247
Mean distance from the main lesion to the inner periphery of the most distant satellite nodule (mm) (±SD)	1.2 (±1.2)	0.7 (±0.5)	0.412
Mean size of the satellite nodules (mm) (±SD)	1.9 (±2.1)	1.7 (±1.1)	0.817
Mean distance from the main lesion to the outer periphery of the most distant satellite nodule (mm)	3.1 (±2.3)	2.4 (±1.4)	0.516

## Data Availability

The data presented in this study are available on request from the corresponding author.
